# High Manifestations of Mental Distress in Arabic Asylum Seekers Accommodated in Collective Centers for Refugees in Germany

**DOI:** 10.3390/ijerph14060612

**Published:** 2017-06-07

**Authors:** Ekaterini Georgiadou, Eva Morawa, Yesim Erim

**Affiliations:** Department of Psychosomatic Medicine and Psychotherapy, Friedrich-Alexander University Erlangen-Nürnberg (FAU), Schwabachanlage 6, 91054 Erlangen, Germany; ekaterini.georgiadou@uk-erlangen.de (E.G.); eva.morawa@uk-erlangen.de (E.M.)

**Keywords:** posttraumatic stress disorder, depression, anxiety, asylum seekers, collective accommodation centers, refugees

## Abstract

The number of asylum seekers in Germany has significantly increased in the last two years. Coming from regions of political conflict and war, the refugees have often experienced traumatic events which designate them as a high risk group for mental disorders. In a sample of Arabic speaking asylum seekers in collective accommodation centers in Erlangen, Germany, we estimated the extent of posttraumatic stress, depression, and anxiety. A further objective of the study was to test the comprehensibility and cultural appropriateness of the Arabic translations of the questionnaires in this sample. Between August and September 2016, 56 Arabic speaking asylum seekers placed among three collective accommodation centers in Erlangen completed self-report questionnaires assessing posttraumatic stress disorder (Essen Trauma-Inventory, ETI), and symptoms of depression (Patient Health Questionnaire—depression module, PHQ-9) and anxiety (Generalized Anxiety Disorder, GAD-7). The prevalence of participants with personally and/or witnessed traumatic events was 80.4% (*n* = 45). About one-third of the examinees (35.7%, *n* = 20) endorsed symptoms of PTSD (posttraumatic stress disorder). The total score for depression in this sample was M = 11.9 (SD = 7.9, range: 0–27). Moderate to severe depression (PHQ-9 score ≥ 15) was found in 35.7% (*n* = 20) of our sample and severe depression (PHQ-9 score ≥ 20) was found in 23.2% (*n* = 13). The total score for anxiety was M = 8.8 (SD = 6.9, range: 0–21), with 26.8% (*n* = 15) of the sample showing symptoms of severe anxiety (GAD-7 score ≥ 15). No significant difference between women and men with respect to frequency and symptom scores of PTSD, depression, and anxiety was found. Amongst asylum seekers of the presented sample, the rates of traumatic events as well as the prevalence of possible PTSD, depression, and anxiety were significantly higher than in the German population. This indicates that the refugee population is in need of culturally sensitive psychological interventions. However, more studies are required to improve the understanding of mental health among this particularly vulnerable population.

## 1. Introduction

By the end of 2015, 65.3 million individuals were forcibly displaced worldwide. About 7% (4.9 million) of all refugees worldwide came from Syria [1]. Over the last two years, Germany and Europe have been witnessing the largest migration recorded in their recent history. Compared with 2014, the number of asylum applications in Germany increased by 155.3% in 2016. In 2015, 441,899 asylum applications (30.8% women) and 1,091,894 entrances of refugees were documented. Most asylum requests came from Syria (35.9%; 158,657) and 6.9% (29,784) were from Iraq. Over fifteen percent (15.3%) of the applications were recorded in Bavaria [2]. In 2016, the number of applications even reached 722,370 [3]. Most of the requests came consistently from Arabic speaking countries (Syria 36.9%; Iraq 6.9%).

Migrants, in general, are a heterogeneous group and the prevalence rates of mental disorders vary widely between studies [4]. The majority of studies with migrants ascertained higher prevalence rates of mental disorders [5] as well as higher symptom scores [6,7,8] in comparison to the German population. In a systematic overview of Lindert et al. [4], the prevalence rates for refugees were higher than those for labor migrants. Prevalence rates of posttraumatic stress disorder (PTSD) vary widely for refugees, between 4% and 86%, for depression between 3% and 81%, and for anxiety between 5% and 90% [4].

Heterogeneous findings have also been reported about the mental health of refugees who are resettled in Germany. Gäbel et al. [9] found symptoms of PTSD in 40% of the 40 participants from 13 different countries. In 1999, Traue et al. [10] examined 99 refugees from Kosovo. Symptoms of PTSD were detected in 42% of the participants, while depression and anxiety were even more frequent, with 80% of the participants manifesting symptoms. In a survey on Russian and ex-Yugoslavian descent refugees [11], at least one mental disorder was present in only 3.5% of the refugees. This outcome was explained as an under-diagnosis by physicians with lacking psychiatric expertise.

Few studies have investigated the mental health problems of the Arabic speaking refugees in the recent years. In a sample of 310 Syrian refugees in Lebanon, the prevalence of current depression was 43.9% [12]. In a sample of 352 refugees from Syria, Alpak et al. [13] reported a frequency of PTSD of 33.5%. They found positive correlations between PTSD, number of traumatic events experienced, and female gender. Kazour et al. [14] found a lifetime prevalence of 35.4% and a point prevalence of 27.2% for PTSD in Syrian refugees in Lebanon. In a survey among Iraqi refugees resettled in the United States, the prevalence of emotional distress, anxiety, and depression was approximately 50% and the risk for PTSD about 31% [15]. In a sample of 225 Iraqi refugees resettled in western Sydney with a mean duration of stay in Australia of 59 months, Slewa-Younan et al. [16] measured PTSD symptoms. Ninety-five percent of participants reported having experienced one or more potentially traumatic event, with a mean of 14.28 events (SD = 8.69). Thirty-one percent of participants met the criteria for clinically significant PTSD symptomatology.

So far, only a few studies have investigated the mental health consequences of refugees from the more recent migration movement in Germany. Richter et al. [17] ascertained one or more mental disorders in 64% of 283 refugees from different countries (mostly from Iran, Russia, Afghanistan, and Iraq). Most frequently they found PTSD (33%) and depression (22%) symptoms. In a cohort of Syrian refugees, the prevalence of depression, examined with Patient Health Questionnaire (PHQ)-4, was 20%, corresponding to results for the German population with the same questionnaire [18]. The prevalence of generalized anxiety was moderately higher in the Syrian refugee sample (19.3%). However, they assume an underrepresentation of the prevalence rates because a high number of men (80%) were included in their study. Also, the prevalence rates could have been underestimated due to avoidance reactions by highly distressed refugees who did not participate in this survey. During the significantly increased arrival of refugees in the summer of 2015, Kröger et al. [19] estimated the extent of posttraumatic stress and depressive symptoms amongst adult asylum seekers from different regions (Balkan States, the Middle East, Northern Africa, rest of Africa) in Braunschweig, Germany. Participants reported high total numbers of traumatic events. The prevalence of PTSD was 20.5% (the Middle East) and 23.6% (total sample); while the emergences of a clinical/subclinical depression were 35.9% (the Middle East) and 25.4% (total sample). Compared to the German population, the prevalence of PTSD was significantly higher amongst asylum seekers.

The different results of the studies illustrate the heterogeneity of the refugee population. In the majority of the studies, a representative sample of refugees could not be recruited. The studies also lack comparability, as they used different psychometric instruments. Information on mental health among refugees is insufficient and therefore further research is required [20]. Several Arabic-speaking countries have witnessed long-lasting war. In the last two years, the most applications for asylum in Germany were requested from Arabic speaking people [2,3]. Refugees who came from countries with intense human rights violations have an increased rate of psychopathological symptoms [21] and the exposure to violence is associated with higher rates of trauma-related disorders [22].

The present study aims to determine the frequency of PTSD, depression, and generalized anxiety amongst asylum seekers in collective accommodation centers for refugees in the municipal area of Erlangen. Furthermore, the prevalence of traumatic events (TEs) will be reported. A further objective of the study was to test the comprehensibility and cultural appropriateness of the Arabic translations of the questionnaires in this sample.

## 2. Materials and Methods

### 2.1. Sample

This study was conducted in cooperation with Erlangen City Council and the charitable organization of Arbeiter-Samariter-Bund (ASB; German Samaritan Workers Union). Participants in the present study were Arabic speaking asylum seekers who fled to Germany and were placed in collective accommodation centers for refugees in Erlangen. During the time of this study, they were waiting for their residence permit. The participants were recruited at three different collective accommodation centers in Erlangen (Bavaria) which were assisted by ASB. At the time of this investigation, there were five collective accommodation centers for asylum seekers from the current refugee crisis. One center was just about to be dissolved and the refugees were transferred to other centers. Because of organizational reasons, only accommodation centers carried by ASB were included in the study. Thus, we recruited from the three largest centers in Erlangen. According to ASB, in total, 80 Arabic speaking asylum seekers were settled in these three housings and 67 of them fulfilled the inclusion criteria for this study. The response rate was 83.6%; as such, a total sample of 56 participants was included. During the survey, our team (first author and an Arabic speaking interpreter from the ASB) went door to door in these accommodations and asked all Arabic speaking people for their participation after explaining the purpose of the study and the conditions of the participation. Those willing to participate were given a questionnaire and they were asked to complete the questions in a shared lounge. In the lounges, up to ten asylum seekers completed the questionnaires at the same time. It was not desired that the participants speak to each other while filling the questionnaires. The team members stayed in the room and offered further explanations if needed. The study participants were also asked to note incomprehensible or unusual language usage. These aspects were discussed by the study team and the translators after collecting the questionnaires.

Inclusion criteria for the study were: age of consent (minimum of 18 years), agreement to participate in the study, and good command of the Arabic language (written and spoken). Exclusion criteria were: having already been examined with the same questionnaire, for example, in preparation for the official hearing, active suicidal ideation, and current symptoms of psychotic disorders or intellectual disability. From 11 participants that did not fulfill the inclusion criteria, five were analphabets, one had already been examined with the same questionnaire, and two participants were not in the accommodation centers during the survey. Three participants did not agree to participate in the study.

Data were obtained between August and September 2016. Participation in the study was completely voluntary and written informed consent was obtained from all participants. All study participants received a reimbursement of 10 €. The study was approved by the Ethics Committee of the University of Erlangen-Nürnberg (Project identification code: 93_16 B).

### 2.2. Assessment Instruments

The survey instruments included demographics, migration-related variables, and symptoms of posttraumatic stress, depressive symptoms, and anxiety.

#### 2.2.1. Essen Trauma-Inventory (ETI)

The Essen Trauma-Inventory [23] is a self-rating questionnaire developed to assess potential TEs and PTSD. The translated version of the Essen Trauma-Inventory was obtained from the author, Dr. Sefik Tagay, and was translated from German to Arabic and transculturally adapted. The validation of the German ETI version was carried out on a sample of a total of 952 persons and included healthy subjects (blood donors) in addition to patients with physical and mental illness. It is an economic, reliable, and valid screening instrument that assesses exposure to a broad range of potential TEs. The questionnaire is based on the Posttraumatic Stress Diagnostic Scale [24] and the revised version of the Impact of Event Scale [25] and is related to posttraumatic symptomatology according to DSM-IV (Diagnostic and Statistical Manual of Mental Disorders) [26] diagnostic criteria. ETI was used particularly in previous studies in migrants with Polish origin [27] and Turkish speaking patients [6].

First of all, the participants marked yes or no in the questionnaire if they personally experienced or witnessed a series of potential TEs (e.g., torture, violence, severe accidents). For the worst TE that they experienced, they rated if this triggered an objective threat to life (A1 criterion) as well as a subjective feeling of threat (A2 criterion). Additionally, they rated the 17 items of PTSD symptom list, including intrusion (five items), avoidance (seven items), and hyperarousal (five items). The PTSD symptom list was rated on a 4-point Likert scale that ranged from “does not match at all” (0 points) to “completely matches” (3 points). Clinically apparent PTSD is indicated if the participant experienced a TE, met both criteria (A1 and A2), and the sum score of the PTSD symptom list achieves a cut-off-value of 27. Cronbach’s α in the present study sample was 0.96.

#### 2.2.2. Patient Health Questionnaire—Depression Module (PHQ-9)

Depression was assessed using the 9-item PHQ-9 [28,29] which includes each of the nine DSM-IV [26] criteria for depression. The Arabic version of PHQ-9 questionnaire is available online [30]. The PHQ is a screening instrument for psychiatric case definition in primary care. The items refer to problems within the last 2 weeks. The score of severity of depression varied from 0 (not present at all) to 3 (present nearly every day), yielding a total score between 0 and 27. The scores for PHQ-9 were used to determine the presence of depression and its severity depending on the following score ranges: 10–14 moderate, 15–19 moderate to severe, and 20–27 severe. Cronbach’s α in the present study sample was 0.89.

#### 2.2.3. Generalized Anxiety Disorder (GAD-7)

To assess participants’ symptom severity of generalized anxiety disorder, we administered the 7-item Generalized Anxiety Scale [31,32]. This questionnaire was translated from German to Arabic, back-translated, and commented upon by their respective authors. Each of the seven items was rated on a 4-point Likert scale. Response options were “not at all” = 0, “several days” = 1, “more than half the days” = 2, and “nearly every day” = 3. Therefore, GAD-7 scores range from 0 to 21, with scores of ≥5, ≥10, and ≥15 representing mild, moderate, and severe anxiety symptom levels. In this study’s sample, internal consistency was α = 0.94.

### 2.3. Statistical Analysis

Statistical analyses were conducted using the SPSS 21 (SPSS Inc., Chicago, IL, USA) statistical package. Prevalence rates were calculated based on available cut-off scores for each questionnaire. In addition to descriptive methods, categorical variables including prevalence rates were compared using *χ*^2^-tests. Fisher’s exact test was used if the expected count in one of the cells was less than 5. To calculate differences between groups, we used *t*-tests for continuous variables and chi-square tests for ordinal or dichotomous variables. If variables did not meet the assumption of normality, the comparison between groups was repeated using non-parametric tests (Mann-Whitney *U*-tests). To investigate the relationship between the scores of the assessment instruments and the duration of stay, we calculated Pearson correlations. The significance level for all tests was set at *p* = 0.05.

## 3. Results

### 3.1. Sociodemographic Data

The sample consisted of 20 woman (35.7%) and 36 men (64.3%). The mean age of the participants was 27.2 years (SD = 7.1, range: 18–45). There were no significant age differences between female and male individuals (M = 29.6, SD = 6.4 and M = 26.1, SD = 7.2, *p* = 0.168). Demographics are described in Table 1. Of the 56 participants, 26 (46.4%) were of Iraqi descent and 19 (33.9%) came from Syria. One (1.8%) participant reported having come from Algeria, five from Palestine (8.9%), three declared their identity as Syro-Palestinian, and one as Arabic (1.8%). Seventy-three (73.2%) were Muslims, 5.4% Christians, and 21.4% belonged to other religions. Twenty-six of the participants were parents and had a mean of 2.5 (range: 1–5) children.

The mean duration of the flight journey was 10.3 weeks (SD = 15.6, range: 1–81). The majority (67.9%, *n* = 38) traveled over mainland and by boats and rubber dinghies across the sea. Only 12 (21.4%) refugees fled just over mainland to Germany. For six participants, the escape route is unknown. On a visual scale from 0 (not at all difficult) to 10 (very difficult), the difficulty of the escape was indicated on average 8.2 (SD = 2.7, range: 0–10). The mean duration of stay in Germany was 7.9 months (SD = 3.7, range: 0.5–14).

### 3.2. TEs and Posttraumatic Stress Disorder

The number of participants who personally experienced and/or witnessed TEs was 80.4% (*n* = 45). Distribution of the different TEs is shown in Table 2. There was no significant difference between men and women in the recording of each TE. Thirteen participants (28.9%) had only one TE, almost half of them (48.9%, *n* = 22) had between two and five TEs, and 22.2% (*n* = 10) had more than five TEs. Fifteen of the female (75%) and thirty of the male (83.3%) participants reported at least one TE (χ² = 1.59, *df* = 1, two-tailed Fisher’s exact test *p* = 0.266). The most distressing TE reported was serious accident/explosion (20%, *n* = 9), physical violence by a stranger (11.1%, *n* = 5), and war effort (11.1%, *n* = 5).

About one-third of this sample (35.7%, *n* = 20) endorsed symptoms of PTSD. There was no significant difference between women and men with respect to PTSD prevalence (40% vs. 33.3%, χ² = 0.25, *df* = 1, *p* = 0.618) and in total score on the PTSD (M = 33.6, SD = 6.9 vs. M = 33.9, SD = 4.9, t_(18)_ = −0.11, *p* = 0.913).

### 3.3. Prevalence and Severity of Depression Symptoms and Anxiety

The total score for depression in this sample was M = 11.9 (SD = 7.9, range: 0–27); there was no significant difference between women and men (M = 13.8, SD = 8.8 vs. M = 10.9, SD = 7.2, t_(54)_ = 1.330, *p* = 0.189). The mean total score for anxiety was M = 8.8 (SD = 6.9, range: 0–21); there was no significant difference between women and men (M = 10.6, SD = 7.0 vs. M = 7.8, SD = 6.7, z = 1.453, *p* = 0.166). Prevalence for depression and anxiety in women and men is shown in Table 3.

### 3.4. Number of Mental Disorders

At least one diagnosis was found in 36 asylum seekers (64.3%). Ten asylum seekers (17.9%) were screened positive for one diagnostic category (PTSD or depression or anxiety disorder), 14 asylum seekers (25%) for two diagnostic categories, and 12 asylum seekers (21.4%) for all three diagnostic categories. Of the total sample, 35.7% (*n* = 20) had no mental distress. Distribution of the number of diagnoses of mental distress is shown in Table 4.

### 3.5. Comparison between Asylum Seekers without Mental Distress and Asylum Seekers with Distress in at Least One Diagnostic Category

As can be seen in Table 5, the results of the group comparison suggest differences between asylum seekers without mental distress and asylum seekers with distress in at least one diagnostic category in the experience of at least one TE and the duration of stay in Germany.

### 3.6. Correlation between PTSD, PHQ-9, and GAD-7 Scores and Duration of Stay

Table 6 displays Pearson’s correlations between variables. Higher total scores in ETI symptoms, PHQ-9, and GAD-7 were related to shorter duration of stay in Germany. Total scores of all three measures were positively correlated.

### 3.7. Cultural Validity of Assessment Instruments

To examine the comprehensibility and cultural validity of the assessment instruments, we asked participants to report problematic formulations in the translation to us. All three instruments were well accepted by the participants and their difficulties in understanding or interpreting were very rare. The few questions concerned only the ETI. It was unclear to a few participants whether the PTSD symptom list should be filled only for the worst TE that they experienced. The questionnaire’s items were well understood and there was no need for an improvement or addition to optimize their comprehensibility.

## 4. Discussion

This study examined the prevalence of TEs, and the manifestation of PTSD, and depressive and anxiety symptoms in Arabic speaking asylum seekers who were placed in collective accommodation centers in Erlangen, Germany. The main result of the present study was the identification of high rates of mental distress in Arabic speaking refugees. Also, asylum seekers without mental distress had fewer TEs and a longer duration of stay in Germany in comparison to asylum seekers with distress in at least one diagnostic category. Men and woman seemed to be equal in TEs and prevalence of PTSD, and depressive and anxiety symptoms.

It is well known that the prevalence rates for refugees are higher than for labor migrants, but they vary widely between international studies [4]. In comparison to other studies about refugees from Syria [12,13,14] and Iraq [15,16], we found consistent results. Compared to studies from Germany [17,18,19], we found similar or higher levels of mental distress.

The manifestation of depression and anxiety symptoms were also high in our sample. In general, the prevalence rates in the literature for depression in refugees vary between 3% and 47%, and for anxiety between 5% and 90% [4]. In studies in Germany, the prevalence rates for depression vary between 20% and 80% [10,17,18,19], and for anxiety between 19.3% and 80% [10,18]. In our sample, 57.1% of the asylum seekers endorsed symptoms of at least moderate depression and 39.3% for at least moderate anxiety. Therefore, our findings are consistent with previous research in Germany.

In a systematic overview, Lindert et al. [4] found varying prevalence rates for PTSD in refugees between 4% and 86%. In other studies in Germany, the prevalence rates varied from 20.5% to 42% [9,10,17,19]. In our sample, 35.7% of the asylum seekers endorsed symptoms of PTSD. In comparison to the recent published study by Kröger et al. [19], we found higher rates of PTSD. In this study, the prevalence rate for PTSD were 20.5% in people from the Middle East. The prevalence rates of PTSD could have been underestimated due to avoidance reactions with PTSD and the limited recruitment. However, the samples are not fully comparable. The survey of Kröger et al. [19] included about 16% of the registered asylum-seekers in Lower Saxony, Germany in 2015. They recruited a sample of 280 refugees of different descent; however, only 39 persons originated from the Middle East, with 12.8% women. Moreover, the current housing conditions and the duration of stay of the participants in this study are unclear. Our study is focused on Arabic speaking asylum seekers in collective accommodation centers. We achieved a high response rate of over 80% in the three largest centers in Erlangen (Bavaria), with a high percentage of women (35.7%). However, our results are comparable to those of Richter et al. [17]. In their study, many refugees from Iraq (therefore Arabic speaking persons) were included and they also used ETI to measure PTSD; 32.2% of the participants met the criteria for PTSD.

In the overall picture, in comparison with the general German population, there is a significantly higher rate of mental disorders in refugees. In our sample, 64.3% (*n* = 36) of the asylum seekers had distress in at least one diagnostic category compared to 27.7% in the German general population [33]. In our sample, 80.4% reported at least one TE and 35.7% showed symptoms of PTSD; in contrast, in the German general population, only 23.8% reported at least one TE and 2.9% met the criteria for PTSD [34]. For a moderate depression (PHQ-9 ≥ 10), the rates in adults in Germany are 8.1% (10.2% women; 6.1% men) [35]; in our sample, 57.1% (65% women; 59.4% men) presented moderate depression and 23.2% presented severe depression (PHQ-9 ≥ 20). A moderate anxiety was reported by 39.3% of our sample in contrast to 5.9% of the general German population [36].

The prevalence rates in our study are higher than in the Arabic samples. The review by Tanios et al. [37] showed different rates of anxiety disorders in Arab populations, with 28.2% in Jordan, 16% in Saudi Arabia, 16.7% in Lebanon, and 10% in the United Arab Emirates. In Lebanon, Karam et al. [38] found a 3.4% lifetime prevalence rate of PTSD, 16.7% prevalence rate of anxiety, and 12.6% prevalence rate of mood disorders. The lifetime prevalence of any disorder was 25.8%. In the postconflict area of the Gaza Strip, de Jong et al. [39] reported a lifetime prevalence for PTSD of 17.8%.

We did not find significant differences between female and male individuals in the number of experienced TEs and the degree of PTSD as well as depression symptoms and anxiety. Furthermore, no gender differences between asylum seekers without mental distress and those with distress in at least one diagnostic category were found. Kessler et al. [40] reported that women are more than twice as likely to present lifetime PTSD than men. Contrariwise, Hauffa et al. [34] did not find a significant difference between men and woman in PTSD prevalence in the German population. However, in the German population, women are found to be more anxious than man [36] and depression is diagnosed in women almost twice as frequently as in men [35]. In a review for anxiety disorders in Arabic speaking countries, most studies showed higher prevalence rates in woman than in men [37]. Some recent studies with refugee populations from Arabic speaking countries found a positive correlation between mental disorders and female gender [12,15], while others did not report gender differences [13,18]. In contrast, de Jong et al. [39] found in the Gaza Strip sample that men had more PTSD symptoms than women. In our sample, 64.3% had distress in at least one diagnostic category, approximately half of the sample (46.4%) had two or more diagnostic categories. These results are comparable to those from Richter et al. [17]. In their study, 63.6% of the asylum seekers who settled in collective accommodation centers had at least one psychiatric diagnosis. However, a larger sample of Arabic speaking refugees is needed to confirm our results.

As expected, high prevalence rates of mental disorders were found and significant correlations between all three questionnaires were observed, which confirmed a high comorbidity. The asylum seekers in our study are accommodated in collective housings for refugees, which is an additional stress factor (e.g., because of divided rooms and lack of privacy). They also suffer from the burden of having no clear residence permission. It is well known that stress can have a crucial impact on mental health [41,42]. Higher rates of PTSD are associated with a temporary residence permit [43]. Demiralay and Haasen [42] classify residence permission status and economic conditions (e.g., living conditions) as stress factors for the acculturation process. Nickerson et al. [44] found in their study with refugees from Iraq that post-migration living difficulties are a predictor for PTSD and depression. In our study, a longer duration of stay in Germany was related to a decrease of symptom scores in ETI as well as PHQ-9 and GAD-7. Moreover, asylum seekers with distress in at least one diagnostic category reported a shorter duration of stay in Germany. A possible explanation is that the mental health of some asylum seekers has improved with longer duration of stay in Germany. Morina et al. [45] estimate remission in an average of 44% of individuals with PTSD in a mean observation period of 40 months. However, the remission rates varied between 8% and 89%, whereupon the highest mean of remission rate (60%) was reported in participants with PTSD following the exposure to a natural disaster. According to this, we expect that the symptom levels will decrease within the next months. Laban et al. [46] suggest that residence permission is beneficial for mental health mainly because of the improvement of living difficulties. Putatively, refugees with an expectancy of getting the residence permit soon reported an improvement of their emotional well-being. Resilience factors may help to restrict the emergence of severe psychopathology. Mental health has been considered to be one of the most important factors of successful adaption and resettlement of refugees [47]. Higher acculturation scores are indirectly related to lower scores for depressed mood and promote mental health indirectly by reducing social alienation and, subsequently, lowering family and personal stress [48]. A positive emigration is favored by flexible coping strategies, social support, and optimism [41]. Van Lente et al. [49] showed that positive mental health is predicted by lower levels of loneliness and higher levels of social support in refugees.

In terms of cultural validity, we asked participants to report problematic formulations in the translations to us. The instruments were well accepted by the participants. Difficulties in understanding or interpreting were very rare. We expect that a good linguistical comprehensibility and good conceptual equivalences relate to the acceptability of the concepts of all three measurements. However, there is a need for several culturally adapted and validated questionnaires for Arabic-speaking refugee populations. Research on selected refugees settled in the West may not allow for results to be generalized to the majority of the world’s refugees [39]. Even the native Arabic speaking population has a large variety of cultures, political systems, ethnicities, and religious beliefs [50]. Cultural beliefs affect interpretation of mental symptoms [51]. Cultural validity of health instruments is crucial for improving our understanding and assessment of health. [52]. Vindbjerg et al. [53] assessed the factorial structure of PTSD in an Arabic speaking population of refugees with the Harvard Trauma Questionnaire [54]. The results of their study indicate that the construct of PTSD is valid among this population.

Concerning demographic properties, our sample is comparable to the basic population of asylum seekers in Germany. In 2016, most asylum applications were made by men (65.7%) and 34.3% by woman, and most requests came from Syria (36.9%) and Iraq (6.9%). The majority of applications (47.2%) were made from asylum seekers between 18 and 35 years old [3]. In our sample, 35.7% were women and the mean age of the total sample was 27.2 years (range: 18–45). Moreover, in our study, we included 83.6% of Arabic speaking asylum seekers who were settled among the three biggest collective accommodation centers in Erlangen and fulfilled the inclusion criteria for our study.

Despite the high response rate, our sample is small and our results should be interpreted in the context of certain limitations that should be addressed in future research. First of all, our study recruitment was focused on Arabic speaking asylum seekers who are placed in collective accommodation centers. Therefore, this sample may not accurately reflect the mental health of the Arabic speaking refugee population in other accommodation forms in Germany. A representative sample of the refugee population is crucial for obtaining correct prevalence rates. However, due to the importance of this kind of data, more detailed studies are needed which will include larger samples. These studies should also compare their results to data obtained from Arabic countries. An additional shortcoming of the present investigation is in regard to the lack of information on psychiatric comorbidity other than PTSD, depression, and anxiety. Also, the question of whether depression and anxiety is the consequence of TEs cannot be answered by our data due to the cross-sectional design of our study. Furthermore, the measurements are not validated for refugee populations and the ETI is based on DSM-IV. Updated research based on DSM-V conceptualization of PTSD is necessary.

## 5. Conclusions

The fact that the prevalence rates of mental disorders and scores of mental distress vary widely confirms that asylum seekers are a heterogeneous group concerning their resilience. Since most of the asylum requests in Germany came from Arabic speaking countries, the lacking data on Arabic speaking refugees’ mental health must be urgently obtained, as it is needed for the planning of health services. Future studies should measure not only mental disorders but also resilience factors and analyze the cultural definitions of mental health and illness. This information will allow adequate planning of culturally sensitive health services.

## Figures and Tables

**Table 1:** Sociodemographics.

**Characteristics**	***n***	% *
**Marital status**		
Single	21	37.5
Married	29	51.8
Divorced	2	3.6
Widowed	1	1.8
Other	3	5.4
**Parental status**		
Parent	26	46.4
Not a parent	22	39.3
No data	8	14.3
**Age (year)**		
18–24	16	28.6
25–29	9	16.1
30–34	7	12.5
≥35	4	7.1
No data	20	35.7
	**M (SD)**	**Range**
**Education in years**		
(*n* = 43)	10.2 (3.4)	4–21

* Add-ups may not be equal to total due to rounding.

**Table 2:** Lifetime prevalence of traumatic events of the total sample (*n* = 56).

Traumatic Event	Personally Experienced		Witnessed		Personally Experienced and Witnessed		Personally Experienced and/or Witnessed	
	*n*	%	*n*	%	*n*	%	*n*	%
War effort	16	28.6	1	1.8	3	5.4	23	41.1
Prisoner/hostage	10	17.9	3	5.4	0	0	13	23.2
Torture	9	16.1	2	3.6	0	0	13	23.2
Physical violence (stranger)	16	28.6	5	8.9	4	7.4	25	44.6
Physical violence (acquaintance)	8	14.3	2	3.6	1	1.8	12	21.4
Death of loved one (e.g., homicide)	8	14.3	4	7.1	0	0	15	26.8
Serious accident/explosion	18	32.1	2	3.6	7	12.5	28	50.0
Serious illness	5	8.9	3	5.4	1	1.8	9	16.1
Sexual harassment (stranger)	1	1.8	0	0	0	0	1	1.8
Sexual harassment (acquaintance)	1	1.8	0	0	0	0	1	1.8
Neglect	9	16.1	1	1.8	0	0	13	23.2
Childhood sexual abuse (stranger)	1	1.8	1	1.8	0	0	2	3.8
Childhood sexual abuse (acquaintance)	1	1.8	0	0	0	0	1	1.8
Natural catastrophe	7	12.5	0	0	1	1.8	8	14.3
Other trauma	7	12.5	1	1.8	0	0	9	16.1
At least one traumatic event	36	64.3	14	25.0	7	12.5	45	80.4

**Table 3:** Prevalence of depressive symptoms and anxiety.

Measures	Woman		Man		Comparison *df* = 1		Total	
	*n*	% *	*n*	% *	χ^2^	*p*	*n*	% *
Depression (PHQ-9)								
Total score ≥ 10	13	65.0	19	59.4	0.78	0.376	32	57.1
Total score ≥ 15	10	50.0	10	27.8	2.76	0.096	20	35.7
Total score ≥ 20	7	35.0	6	16.7	2.42	0.186	13	23.2
Anxiety (GAD-7)								
Total score ≥ 10	10	52.6	12	33.3	1.93	0.165	22	39.3
Total score ≥ 15	7	36.8	8	22.2	1.34	0.247	15	26.8

PHQ-9, Patient Health Questionnaire—depression module; GAD-7, Generalized anxiety disorder; * Valid values.

**Table 4:** Distribution of the number of diagnoses of mental distress.

Number of Diagnoses	*n* (%)
Single diagnostic category	
Only PTSD	2 (3.6)
Only depression	7 (12.5)
Only anxiety	1 (1.8)
Two diagnostic categories	
PTSD and depression	5 (8.9)
PTSD and anxiety	1 (1.8)
Depression and anxiety	8 (14.3)
Three diagnostic categories	
PTSD and depression and anxiety	12 (21.4)

PTSD = posttraumatic stress disorder; Depression = Patient Health Questionnaire—depression module total score ≥ 10; Anxiety = Generalized anxiety disorder total score ≥ 10.

**Table 5:** Comparison between asylum seekers without mental distress and asylum seekers with distress in at least one diagnostic category.

Variables	Asylum Seekers without Mental Distress (*n* = 20)		Asylum Seekers with Distress in at Least one Diagnostic Category (*n* = 36)		Comparison	
	*n*	%	*n*	%		*p*
Gender, female	7	35.0	13	36.1	χ^2^_(1)_ = 0.007	0.585
At least one TE	13	65.0	32	88.9	χ^2^_(1)_ = 4.694	0.040
Marital status						
Single	10	50.0	11	30.6	χ^2^_(4)_ = 7.953	0.093
Married	8	40.0	21	58.3		
Divorced	2	10.0	0	0.0		
Widowed	0	0.0	1	2.8		
Other	0	0.0	3	8.3		
	**Mean**	**SD**	**Mean**	**SD**		***p***
Education years	9.61	4.13	10.56	2.87	t_(41)_ = 0.889	0.379
Duration of stay	10.29	2.92	6.63	3.44	t_(49)_ = 3.755	<0.001

TE = Traumatic event.

**Table 6:** Pearson’s correlations between variables.

Measures	PHQ-9 Total	GAD-7 Total	Duration of Stay
ETI-symptoms total	0.641 **	0.652 **	−0.566 **
PHQ-9 total		0.837 **	−0.387 *
GAD-7 total			−0.446 *

ETI, Essen Trauma-Inventory; PHQ-9, Patient Health Questionnaire—depression module; GAD-7, Generalized anxiety disorder. * *p* < 0.01; ** *p* < 0.001.
